# Potential Application of Radiomics for Differentiating Solitary Pulmonary Nodules

**DOI:** 10.4172/2167-7964.1000218

**Published:** 2016-03-21

**Authors:** Kaikai Wei, Huifang Su, Guofeng Zhou, Rong Zhang, Peiqiang Cai, Yi Fan, Chuanmiao Xie, Baowei Fei, Zhenfeng Zhang

**Affiliations:** 1 Sun Yat-sen University Cancer Center, State Key Laboratory of Oncology in South China, Collaborative Innovation Center for Cancer Medicine, Guangzhou, People’s Republic of China; 2 Department of Radiology, Union Hospital, Tongji Medical College, Huazhong University of Science and Technology, Wuhan, People’s Republic of China; 3 Department of Radiation Oncology, Perelman School of Medicine, University of Pennsylvania, Philadelphia, USA; 4 Department of Radiology and Imaging Sciences, Emory University School of Medicine; Department of Biomedical Engineering, Emory University and Georgia Institute of Technology, Atlanta, USA

**Keywords:** Radiomics, Pulmonary nodules, Differentiation

## Abstract

A solitary pulmonary nodule is defined as radiographic lesion with diameters no more than 3 cm and completely surrounded by normal lung tissue. It is commonly encountered in clinical practice and its diagnosis is a big challenge. Medical imaging, as a non-invasive approach, plays a crucial role in the diagnosis of solitary pulmonary nodules since the potential morbidity of surgery and the limits of biopsy. Advanced hardware, image acquisition and analysis technologies have led to the utilization of imaging towards quantitative imaging. With the aim of mining more useful information from image data, radiomics with high-throughput extraction can play a useful role. This article is to introduce the current state of radiomics studies and describe the general procedures. Another objective of this paper is to discover the feasibility and potential of radiomics methods on differentiating solitary pulmonary nodules and to look into the future direction of radiomics in this area.

## Introduction

The term “Radiomics” derives from the combination of word “radiology” and the suffix “omics”. It is a new extension of omics methods applied to quantitative radiology. Radiomics refers to extracting and analyzing features from digital medical images systemically [1]. Segal and his colleagues had carried out one of the pioneer studies on radiomics, which indicated the strong relationship between CT (computed tomography) image traits and gene expression in liver cancer where 78% global gene expression profiles were constructed by the combination of 28 imaging traits [2]. Extraction and analysis approaches provide us the sight of deeply interpretation of image sources. However, that is a retrospective study and the sample size is small. Furthermore, the reliability of imaging traits were all manually assessed, which may introduce an inevitable bias. Due to the development of automatic image segmentation and statistical analysis, reproducibility and stability of lesion delineation was improved. More features and analysis pattern were developed. All these advancements make radiomics and quantitative imaging possible. Another study of 1019 patients showed the correlation between radiomic signature and gene expression in lung or head and neck cancer, suggesting a cost effective access in cancer treatment [3].

**Figure 1:**
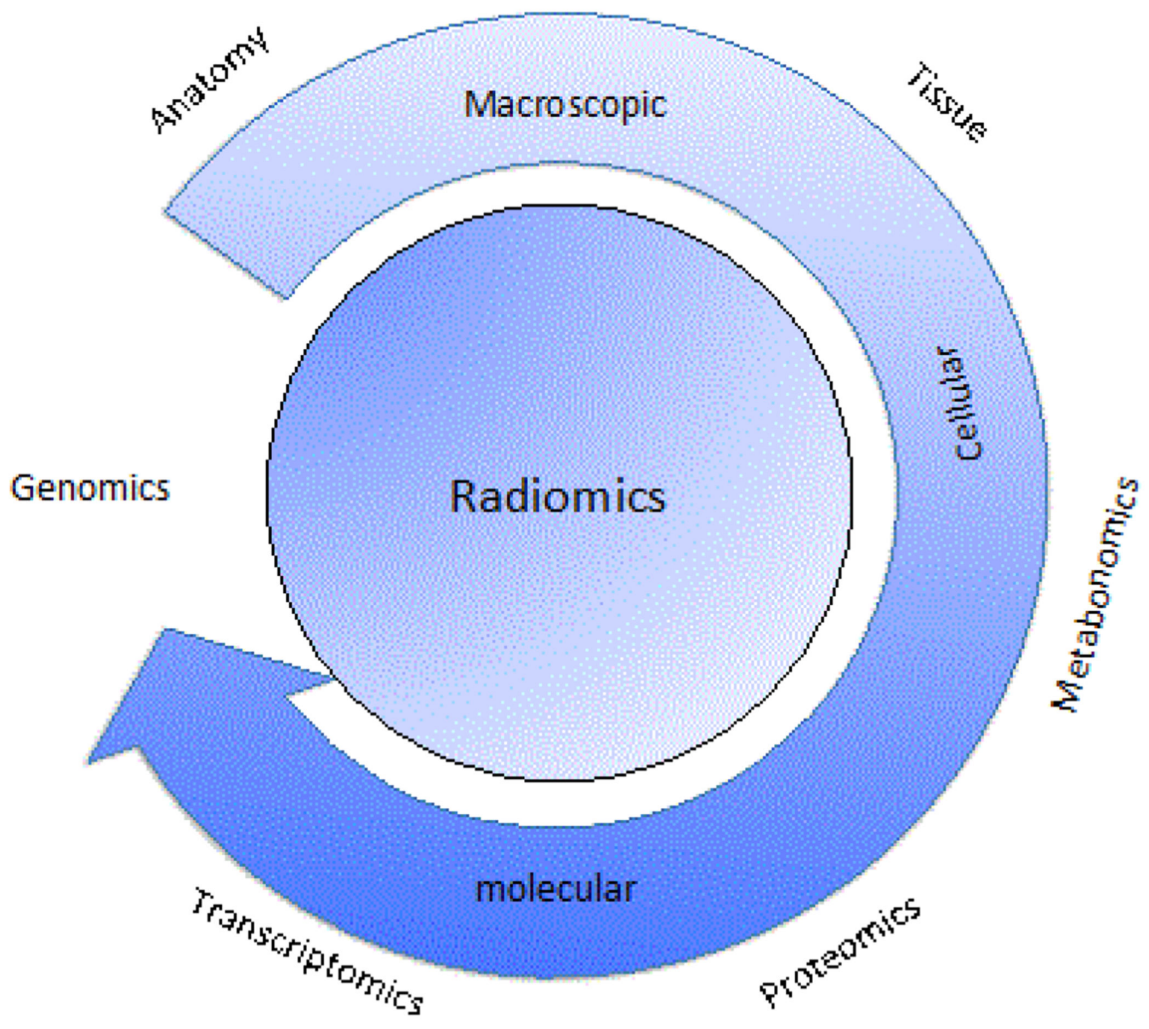
Radiomics is a comprehensive subject that trying to extract information from anatomic structure to molecular structure, it is at the centre of diagnosissurveillances and therapeutic planning.

Solitary pulmonary nodule (SPN) is a common encountered issue in clinical practice. It has been defined as “a rounded or irregular opacity, well or poorly defined measuring up to 3cm in diameter” on CT images [4]. Pretesting possibility of malignancy plays a pivotal role in the management of SPNs. Clinical factors, radiologic features and biopsy are commonly used in distinguishing malignant from benign solitary nodules. When clinical factors, such as location in the upper lobe, were confirmed, the possibility of lung cancer would increase, as shown in an evidence-based prospective study [5]. However, some clinical histories show its limitation, for instance, a smoking history is no longer a factor in the determination of malignancy of pulmonary nodules because of an increasing population of lung adenocarcinoma in younger and non-smoking patients [6]. Biopsy is considered as the “golden standard” for the diagnosis of lung cancer. The applications of biopsy are limited actually in clinic because it is an invasive procedure that may introduce complications after sampling. Radiologic diagnosis based on image features is the most common procedure in clinical practice as a non-invasive approach for disease management. Features, such as margin, size, and calcification, are assessed by radiologists and then integrated into an inclination of malignant disease. Innovations in new imaging technology, contrast agents and standardized protocols leads to improved diagnosis accuracy, which allows radiologist to obtain more useful features and thus improve diagnostic accuracy. The more useful features of lesion we can extract, the higher the diagnostic accuracy we may be able to achieve. In this paper, we discuss the feasibility and potential of radiomics and its application in differentiating solitary pulmonary nodules (Figure 1).

## Image Data and Analysis Tools for Radiomics Studies

Data acquisition and analysis methodology are two core elements in a radiomics study. Data mainly contains image data and clinical data, whereas analysis tools are used to extract features and perform statistical analysis. One destination of a radiomics study is to provide prognostic prediction or treatment planning. Feature extraction methods should be reproducible and robust for lesion delineation. One study of 32 patients showed that 29 features were reproducible among 329 image features [7]. Another study of 56 patients investigated the reproducibility of quantitative image features among 3 different CT scanners and the results indicated that 138 out of 328 features were reproducible [8]. Assessing tools are important for radiomics study. Area under the curve (AUC), receiver operating characteristic (ROC) curves, and concordance index (CI) have been used to assess the reproducibility of features and the performance of models.

Images from different modalities are imported into software systems for segmentation and feature extraction. Software tools allow operators to segment the images manually, semi-automatically or automatically. Region of interest (ROI) from images is further processed for digital feature analysis [9,10]. Large amounts of features can be studied and more features can be added into the analysis. Some features are expressed as numerical patterns. For example, texture feature, which has been widely used in computer science, has been applied in radiomics. Features need to be independent, informative, stable, and reproducible. These characteristics are important for prediction or diagnosis models [7,11-13]. Meanwhile, advanced algorithms are capable of handling large amounts of features and selecting stable, reproducible, independent, and informative ones. For example, mRMR (minimum redundancy maximum relevance) feature selection is an algorithm that gained considerable improvement in feature selection and classification accuracy and has been used in radiomics research [14,15]. Extensive features and optimal algorithms play a critical role in the development of radiomics. Therefore, the collaboration between medical experts and computer engineers is also important in a radiomics study.

## Radiomics Studies in Cancer

One of the early works was to decode gene expression by exploring radiographic features in liver cancer [2]. The trial showed that the gene expression profiles of hepatic carcinoma were associated with their radiographic features, suggesting that the features extracted from the images were able to reflect gene expressions in a convoluted manner. More radiomics studies on cancer were performed after the very first attempt of radiomics in liver cancer. Those studies were first focused on the molecular aspect and were to decode gene expression by radiomics. More studies were carried out in preclinical and clinical medicine [16], and the relationship between gene pattern or expression and medical imaging were investigated [2, 3].

Medical images are collected retrospectively and investigated with patients’ information as well as follow-up data. These studies are to demonstrate the relationship between imaging features and information with directly and indirectly significant benefits for diagnosis or therapy, in the purpose of developing a number of objective and robust features as descriptors for potential use in clinic. Various features have been validated to be associated with biological information ranging from genes to tissue in various diseases. For instance, image features were demonstrated to reveal the association with gene expression either in expression levels or patterns in liver cancer, lung cancer, head and neck cancer [2, 17]. The same results also suggested that there is a relationship between features and heterogeneity in lung and liver cancers [18]. Those micro- invisible characteristics may directly associate with overall survival, disease development and responses to treatments. It has been demonstrated that overall survival was related closely to image features in lung, breast, and colorectal cancer [19-21]. Recently it was attested that some imaging features strongly predict the distant metastasis of adenocarcinoma after radiotherapy [14], and radiomics-based features were also studied to be associated with pneumonitis development or metastasis after radiotherapy [22]. However, most of these researches are retrospective and with a small sample size, more evidence should be provided by large scale perspective studies.

As for response to therapies, most studies focused on drug response while in fact there are more we can do. For instance, radiotherapy dose can be linked with radiomics-based features [22, 23]. All these indicated the potential ability of radiomics features as biomarkers in predicting and surveillance of diseases; pioneer studies showed encouraging results [24]. The extensive application of radiomics prospects a bright future. Besides, we also notice those different modalities and their image acquisitions, including image data of CT, MRI (magnetic resonance image), and PET (positron emission tomography), provide us various approaches to reprocess data sources. By employing appropriate processing methods, we may make the best use of these data sources. We also notice that radiomics research on cancer is no longer retrospective, but also extended its range to animal experimental studies and perspective clinical tials [25], providing a strong evidence of the great potential of radiomics.

## Potential and feasibility of radiomics on diagnosis of solitary pulmonary nodules

The The application of radiomics for differentiating solitary pulmonary nodules is to find a series of characteristic features, which would show the significant differences between malignant and benign lesions. We hypothesize that radiomics is capable for differentiating the probability of malignancy of solitary pulmonary nodules because of the following reasons: first and foremost, malignant and benign nodules are totally different either in cell morphology or biology behavior. Since radiomics has been considered to have the potential on distinguishing different phenotypes of cancer, the differences between malignant nodules and benign ones are therefore supposed to be revealed by radiomics features. Second, the high contrast between pulmonary nodules and lung parenchyma makes it a natural advantage in lung nodule segmentation. Many powerful segmentation algorithms are proposed, some of which are quite promising [26]. A robust segmentation algorithm is required for reproducibility. An automatic or semi-automatic segmentation can save time, which makes it more usable if it is used in clinical practice. We have recently developed a superpixel-base novel segmentation method [27] and are trying to apply for precise segmentation of SPNs. Third, more and more features have been proposed and various decision models are developed to handle massive high-dimension data [28]. These powerful tools are designed to interpret data that cannot be comprehended by human in quantity or dimension.

A study of radiomics on pulmonary nodules has been performed and the extracted parameters such as texture features on CT were proved useful in differentiating transient from persistent part-solid nodules [29]. More features are being studied and confirmed to be significant in different diseases and may be applied to a radiomics study of SPNs [30].

Most radiomics studies related to lung disease have been performed on the basis of CT images. Studies on reproducibility of radiomics features on images from different CT scanners suggest the potential ability of radiomics to integrate information across machines [7, 8]. A few studies on MR or PET showed positive results indicating the ability of radiomics on processing information across modalities [31, 32]. MRI is known to have the advantage of providing not only anatomic but also functional images, compared with CT, and the reprocess of functional images may offer clinicians more useful information about metabolism, which would provide complementary information to improve diagnosis accuracy.

Although various studies on MRI or PET are performed for the detection of pulmonary nodules, CT images are recognized as the gold standard; most lung nodules are first detected with CT, especially in recent popular lung screen programs where low-dose CT screening has led to an increasing amount of image data and thus confront us a challenge but also a chance for radiomics studies of SPNs [33]. Differentiating nodules between malignant and benign ones is a key issue since patients with malignant pulmonary nodules will get a better prognosis or even be cured under earlier intervention, and it can also supply us with valuable information to the final protocol so as to confirm the different treatment decision of lung nodules. For nodules that are no longer suitable for resection, monitoring the progression of the lesions after treatment becomes the first option to consider. Lots of traditional radiological diagnosis studies were performed on this subject (Table1), but their limitations are also obvious [34]. For instance, we used to differentiating lung nodules by features such as morphology, size, density/intensity, and some of which are evaluated by radiologists manually and subjectively. These subjective evaluations inevitably introduce bias. Another question is how we process these features and make them accurate predictors. To date, different statistic models have been utilized by using various features to predict the malignancies of pulmonary nodules [35]. Features including clinical data, location, size (e.g., diameter, doubling time, volume), densities, contrast enhancement, certain significant signs (e.g., halo sign) and functional features (e.g., FDG/PET SUV, MRI ADC) were used as valuable variances in these models [36-38]. We believe that radiomics is an adaption to the emergence of more detailed accurate classification and individualized treatments for SPNs.

## Conclusion

Radiomics requires a combination of radiology, bioinformatics, and biomedical engineering, applies imaging features as elemental units, and utilizes different algorithms and models as tools to analyse and transform these units into useful, readable and reliable diagnostic information. Its emergence brings a new way in understanding the etiology, pathology and progression of disease. Radiomics-based accurate and detailed information extracted from digital medical images is correlated with disease characteristics, and can be widely applied to diagnosis, surveillance, and treatment planning of malignant diseases. Differentiating malignant lung nodules is a common and crucial problem in routine clinical practices. Radiomics features with their high association with cellular and molecular characteristics of lesions have been proved to be useful in differentiating pulmonary nodules, and a number of features have been discovered and classified in assessing malignancy of lung nodules [13]. More excitingly, the number of such features is increasing and more stable informative features are convinced to be closely correlated with the development and progression of disease. However, difficulties still exist down the road to effectively integrate and analyse those massive medical data. Firstly, different machines or protocols among hospitals may lead to the bias of medical image quality. In order to solve these problems, globally approved standards on scanning protocols have to be established, which will aim to minimize the intra- and inter-device variation and thus to improve the reproducibility and stability. Secondly, radiomics studies depend on various algorithms, statistic methods and tests, but the processing of data is often simple due to lack of expertise in statistics, for example, many studies were deemed to inappropriately apply tests of significance [45]. Proper application of these can improve the credibility of the results before they are to be finally validated in clinical practices. Refining the large amount of information is another issue since they contain lots of both useful and useless information. Thirdly, although features extracted from images have been found to be related to molecular phenotype or clinical outcomes, how to illustrate this relationship leaves a huge room for further investigation [43]. Certain feature signatures are explained to be correlated with clinical characteristics, but the value of other features remains unknown. Thus, efforts from both clinical experts and professionals from other fields such as mathematics still need to be made to untangle those issues in the long term.

## Future Perspectives

The advent of radiomics is more than an emerge of an available tools, but a potential revolution in medicine through digging valuable medical information by computer technologies to improve our health and life quality. Appropriate algorithm and computer intelligence with powerful processing ability allow us to make more accurate and effective diagnosis and therapeutic plan for cancer than ever before.

The potential application of radiomics is therefore feasible and invaluable. Hundreds of thousands of medical image data is being created every second in every department of radiology all over the world, which supports us with a great number of data that contains valuable source information for radiomics studies, which can help determine malignancy of a tumor and even decipher its gene expression. Moreover, radiomics provides a unique approach and insight that render us to judge the cancer as a whole, to view, and to interpret biological behaviour or characteristics of the body tissues or organs. For example, heterogeneity caused by a large amount of genetic diversity within a tumor or among tumours of the same patient is thought to play a weighty role in cancer growing pathway and results in phenotypic variations, which brings further challenges in cancer individualized precision therapy. It has been demonstrated that heterogeneity, which reveals biology characteristics, drug-response and disease prognosis, is correlated with medical images by radiomics analysis [46].

In summary, the development of experimental medical science along with radiomics will give us the opportunity to interpret more diagnostic information from imaging and thus improve the use of medical imaging and make it a more powerful tool that can guide the clinical procedures in diagnosis, surveillance or prognosis.

## Figures and Tables

**Table 1:** Radiographic characteristics used in differentiating pulmonary nodules.

Characteristics	Modalities	Conclusion	Reference
Size Growth Rate	CT CT	Nodule size larger than 2 cm in diameter having a higher rate of malignancy than smaller nodules Pulmonary nodules with high growth rate were proved to be malignant	MacMahon et al. [42] Ko et al. [43]
Morphology Margin Shape Calcification	CT CT	A nodule with an irregular or speculate margin with distortion of adjacent vessels is likely to be malignant, while one with smooth and well defined is usually benign Calcification is recognized as benign pattern of pulmonary nodules Higher prevalence of malignancy among nonsolid and part-solid nodules than among solid nodules	Erasmus et al. [44]
Attenuation Solid Non-solid Part-solid SUV_max_ DWI*	CT FDG-ET* MRI	Higher SUV_max*_ indicates more probability of being malignant ADC* value of benign lesions was statistically higher than that of malignant tumours LSR* evaluation was useful and practical	Ohno et al. [25] Liu et al. [45] Koyama et al. [46]

* DWI: Diffusion Weighted Imaging; FDG-PET: Fluorodeoxyglucose-Position Emission Tomography; ADC: Apparent Diffusion Coefficient; SUV: Standardized Uptake Value; LSR: signal–intensity ratios between lesion and spinal cord.
